# Genotype–phenotype correlations in *DSP*-associated arrhythmogenic cardiomyopathy that initially presents as myocarditis: a case report and literature review

**DOI:** 10.3389/fcvm.2026.1789765

**Published:** 2026-05-19

**Authors:** Xinyu Lin, Dequan Su, Shubin Lin, Fang Yang

**Affiliations:** 1 Department of Cardiology, Fudan University Affiliated Children’s Hospital Xiamen Hospital (Xiamen Children’s Hospital), Xiamen City, Fujian Province, China; 2 Department of Urology, Fudan University Affiliated Children’s Hospital Xiamen Hospital (Xiamen Children’s Hospital), Xiamen City, Fujian Province, China; 3 Department of Pediatrics, Provincial Clinical Medical College of Fujian Medical University (Fuzhou University Affiliated Provincial Hospital, Fujian Provincial Hospital), Fuzhou, Fujian, China

**Keywords:** arrhythmogenic cardiomyopathy, bioinformatics analysis, desmoplakin, literature review, mutation, myocarditis

## Abstract

**Objective:**

This case report summarizes the clinical characteristics and molecular genetic features of arrhythmogenic cardiomyopathy (ACM) caused by mutations in *DSP* that initially presented as myocarditis.

**Methods:**

The clinical manifestations, genetic variation characteristics, and prognosis of a case that presented with an episode of myocarditis and was ultimately diagnosed with ACM caused by compound heterozygous variants in the *DSP* gene were retrospectively analyzed. Pubmed, Medline, Web of Science, China National Knowledge Infrastructure, and Wanfang Database were searched to summarize the clinical characteristics of ACM caused by mutations in *DSP* that initially presents as myocarditis and to explore the relationship between genetics and phenotypes.

**Results:**

A 9-year-old female patient was diagnosed with myocarditis due to recurrent dizziness, elevated troponin levels, frequent premature ventricular contractions, and imaging findings. Whole-exome sequencing (WES) identified compound heterozygous variants in the *DSP* gene (NM_004415.4) in the proband, namely, c.7964C > G and c.6785G > T, which were inherited from her father and mother, respectively. A total of 22 cases with *DSP* gene mutations were identified, including 13 heterozygous mutations, nine nonsense mutations, six frameshift mutations, five missense mutations, one homozygous mutation, one splice-site mutation, and one with compound heterozygous variants. All the patients were ultimately diagnosed with ACM and initially presented with myocarditis. Troponin levels were elevated in all patients. Electrocardiograms showed abnormalities in all cases, including ST-T changes in nine cases, ventricular tachycardia in seven cases, premature ventricular contractions in two cases, and low voltage in two cases. Echocardiography demonstrated left ventricular structural abnormalities or dysfunction in 13 cases, right ventricular structural abnormalities or dysfunction in one case, and biventricular structural abnormalities or dysfunction in one case.

**Conclusion:**

ACM caused by *DSP* variants often presents with recurrent episodes of myocarditis, accompanied by elevated troponin levels, an abnormal electrocardiogram, and changes in cardiac structure and function. Gene detection results provide a basis for clinical diagnosis. This study found novel compound heterozygous variants in the *DSP* gene and expanded the clinical feature spectrum of *DSP*-associated ACM.

## 1 Introduction

Arrhythmogenic cardiomyopathy (ACM) is an inherited heart muscle disease with a prevalence ranging from 1:5,000 to 1:2,500 (1). It is a progressive, chronic familial disease mainly characterized by ventricular arrhythmias, syncope, heart failure, and sudden cardiac death (SCD). Between 30% and 60% of patients have a positive family history, with the majority of cases following an autosomal dominant inheritance pattern. The phenotype is incompletely penetrant, and the expression rate varies depending on the genetic mutation (2). Autosomal recessive inheritance has also been reported (3). The ClinVar database shows sufficient evidence for the dosage pathogenicity of the *DSP* gene. Multi-gene mutations are associated with a higher risk of arrhythmias compared to single-gene mutations (4). According to the Fuwai classification, the majority of *DSP* mutations are classified as type 3; ventricular arrhythmias are common, and the disease usually develops progressively, accompanied by severe left ventricular dysfunction, and can progress to end-stage heart failure. Histological examination shows biventricular involvement and obvious fibrofatty tissue infiltration. The left ventricle is more severe, usually involving the inferior wall, and the right ventricle has moderate fibrofatty infiltration (5, 6). The majority of the known pathogenic variants associated with ACM are found in desmoplakin (DSP), the largest desmosomal protein. This article reports a case of ACM caused by compound heterozygous variants in the *DSP* gene [NM_004415.4] and summarizes the relationship between the genotype and phenotype of *DSP*-associated ACM that initially presents as myocarditis, to provide a reference for the early identification, diagnosis, prognosis, and genetic counseling of the disease.

## 2 Materials and methods

### 2.1 Subjects

In this retrospective study, a child with ACM caused by *DSP* gene mutations, who was admitted to Fujian Provincial Hospital in February 2022, was enrolled as the research subject. The clinical data of the patient, including gender, age, medical history, clinical manifestations, laboratory examinations, and genetic testing results, were collected. This study was approved by the Ethics Committee of Fujian Provincial Hospital (No. K2024-12-058), and all the guardians of the participating family members signed the informed consent form.

### 2.2 Whole-exome sequencing

Whole-exome sequencing of the proband was performed, and custom-made capture probes containing the whole exon regions were used to capture the target gene. The data were compared with the hg38 human reference genome sequence. Sanger sequencing was used for verification, and primers for the target sequences were designed according to the verified site sequence of the *DSP* gene. The suspected mutation site, *DSP* [NM_004415.4:c.6785G > T(p.Gly2262Val)], was amplified by PCR for 30 cycles. The following primers were used: forward, CCACATACTGGTCTGCTCTTGCT; reverse, TAGGCTTCCTCCACTGGTAACCT. For *DSP* (NM_004415.4):c.7964C > G(p.Ala2655Gly), the same amplification method was used with the following primers: forward, ACAAGGGCCTTGTTGACAGGAA; reverse, AAGGCTTTCTGAGCAGGCTTCA. The annealing temperature was set to 60 °C. The primers were synthesized by the Suzhou Semecco Gene Technology Laboratory, and the PCR products were sequenced using an ABI 3730XL sequencer (SeqGen, CA, USA).

### 2.3 Bioinformatics analysis methods

**Figure 1:**
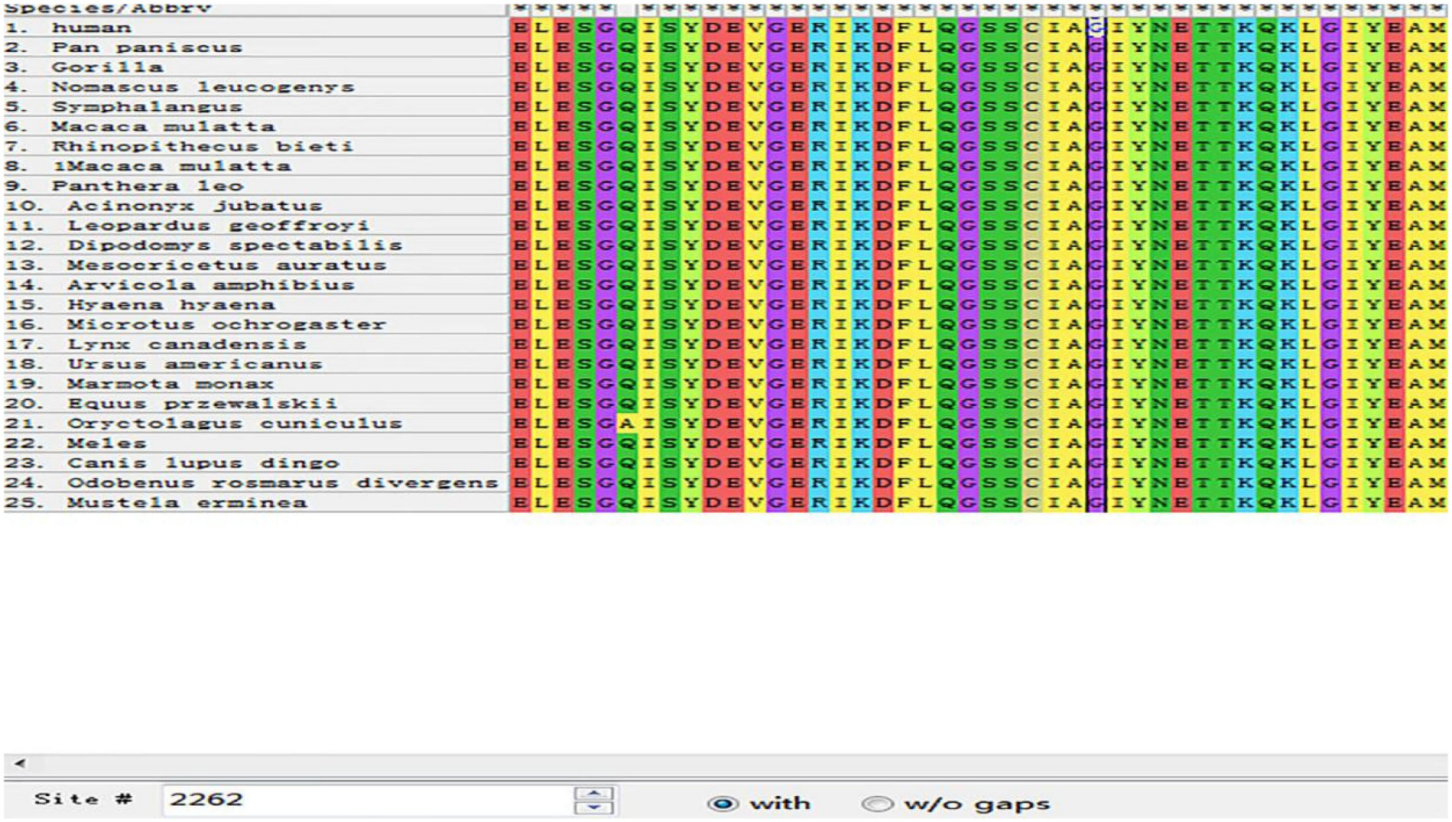
Analysis of the conservation of amino acid sequence near G2262V (black box shows the mutation site of p.Gly2262Val).

**Figure 2:**
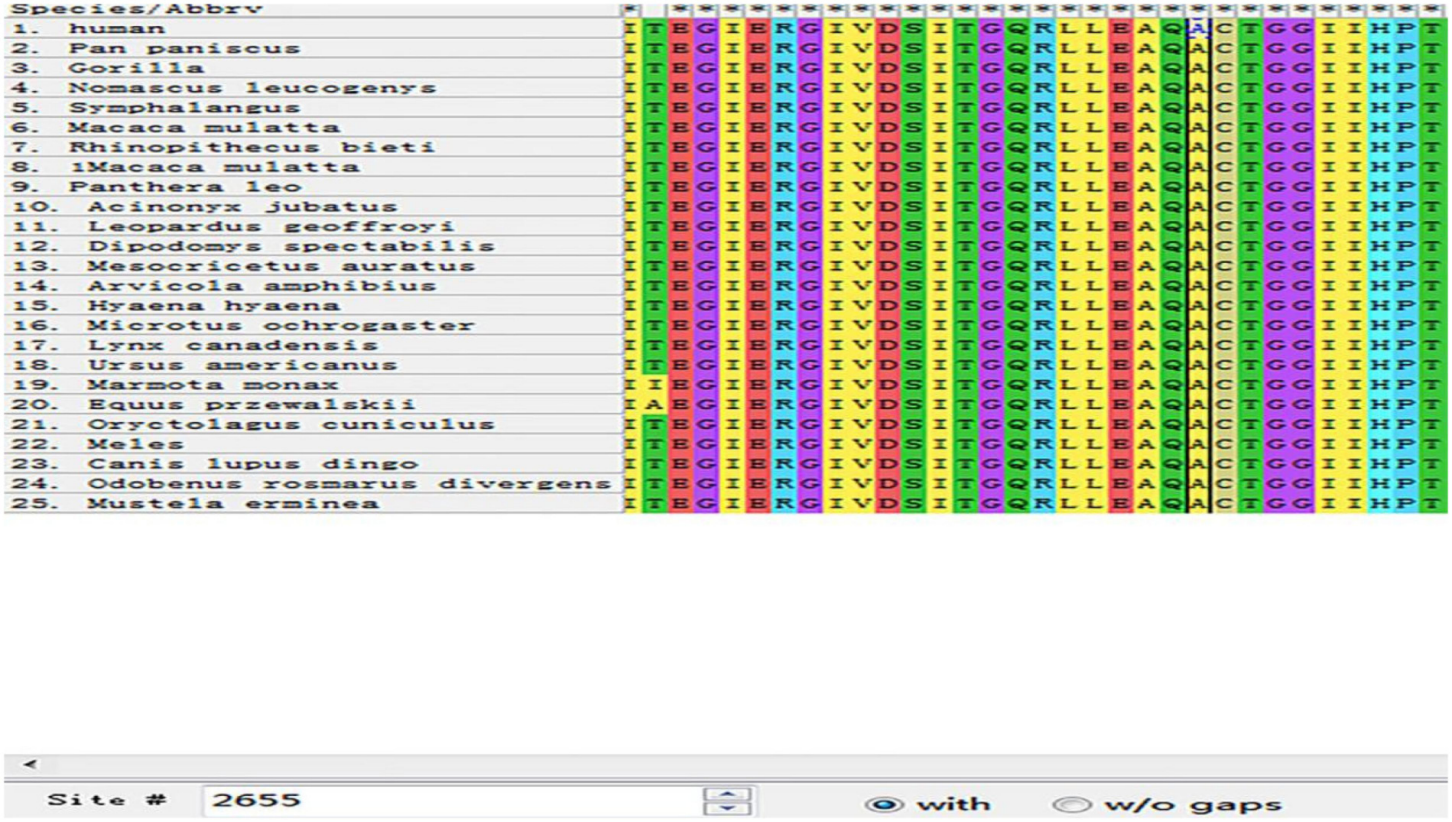
Analysis of the conservation of amino acid sequence near A2655G (black box shows the mutation site of p.Ala2655Gly).

**Figure 3:**
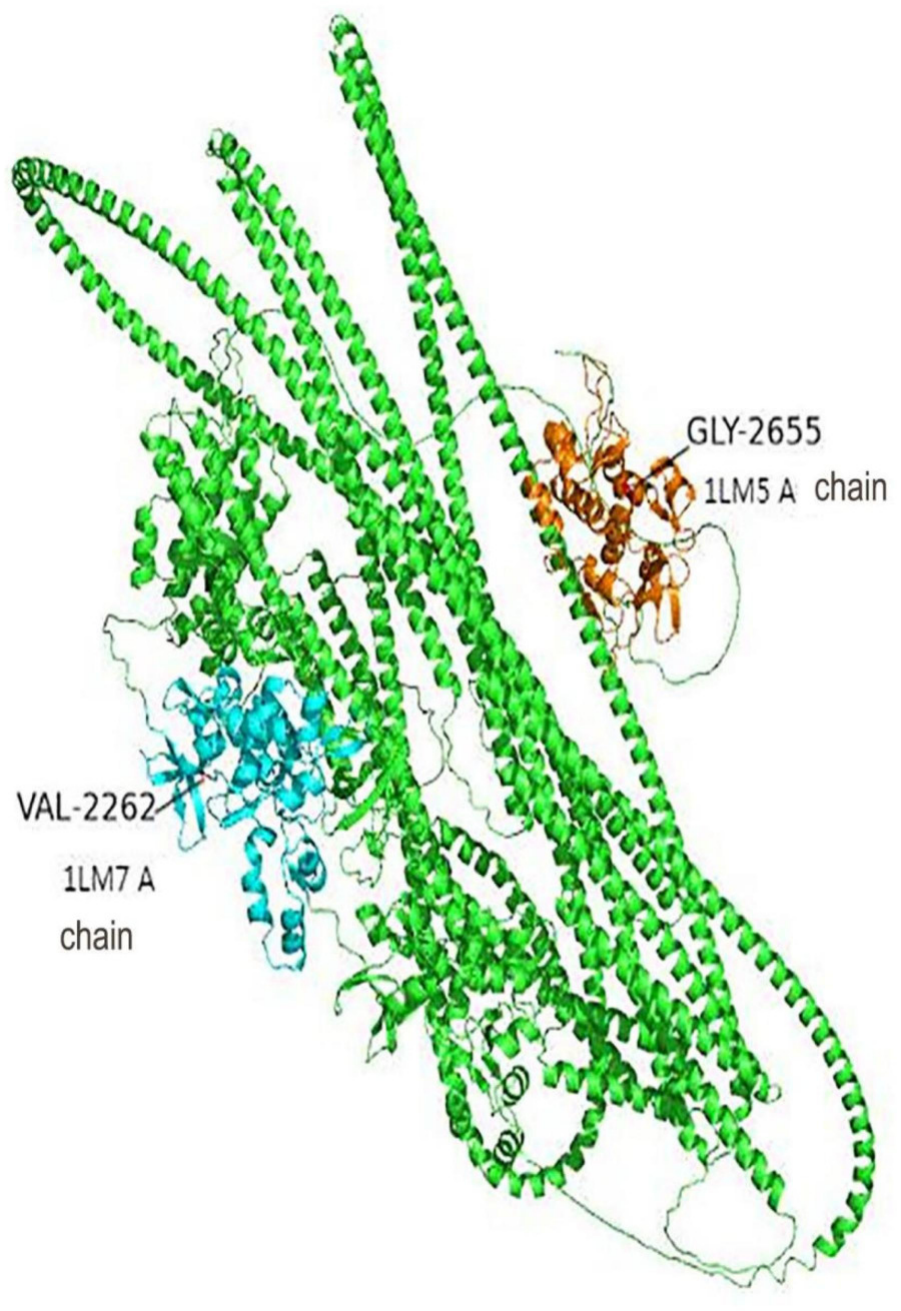
The structure of desmoplakin after mutation. p.Gly2262Val is located at the A chain of crystal structure 1LM7 (blue part). p.Ala2655Gly is located at the A chain of crystal structure 1LM5 (orange part).

**Figure 4:**
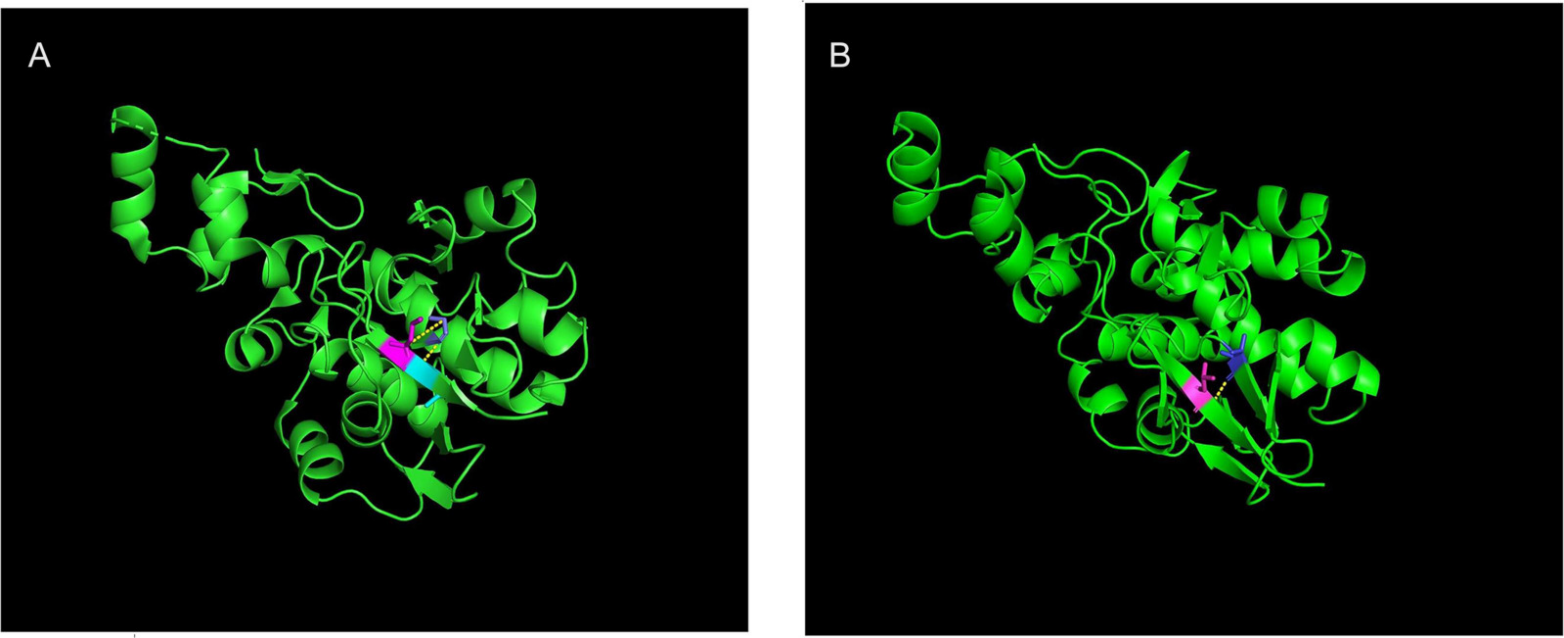
Prediction of changes in the tertiary structure of 1LM7 after p.G2262V mutation (dark blue) by Alphafold3 (https://alphafoldserver.com/). **(A)** DSP G2262; **(B)** DSP V2262.

**Figure 5:**
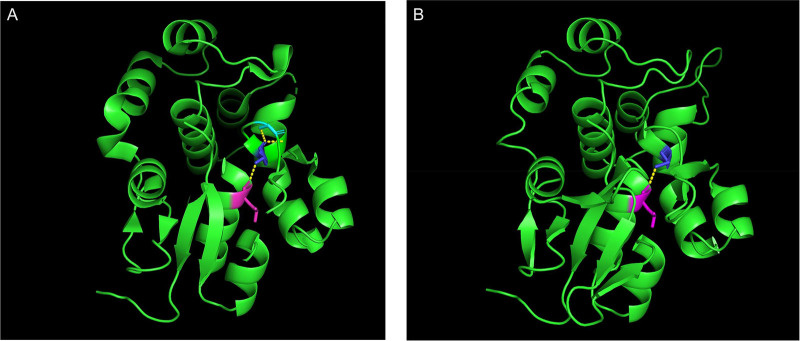
Prediction of changes in the tertiary structure of 1LM5 after p.A2655G mutation (dark blue) by Alphafold3 (https://alphafoldserver.com/). **(A)** DSP A2655.5; **(B)** DSP G2655.

MEGA11 software was used to analyze the conservation of amino acid sequences near G2262V and A2655G by ClustalW. After aligning orthologous sequences from other species, it was observed that Gly2262 and Ala2655 in DSP were highly conserved in multiple species (Figures 1, 2). The p.Gly2262Val mutation was located in the crystal structure of 1LM7, and the p.Ala2655Gly mutation was located in the crystal structure of 1LM5. The AlphaFold3 protein structure database (https://alphafoldserver.com/) was used to predict the tertiary structure of the protein after the compound heterozygous variants of the *DSP* gene (NM_004415.4) (Figure 3), and Pymol was used to analyze the interaction forces between the mutated amino acids and the surrounding residues. The number and length of the hydrogen bonds around the mutated amino acids were changed (Figures 4, 5).

## 3 Results

### 3.1 Phenotypes and genotypes of the reported case

**Figure 6:**
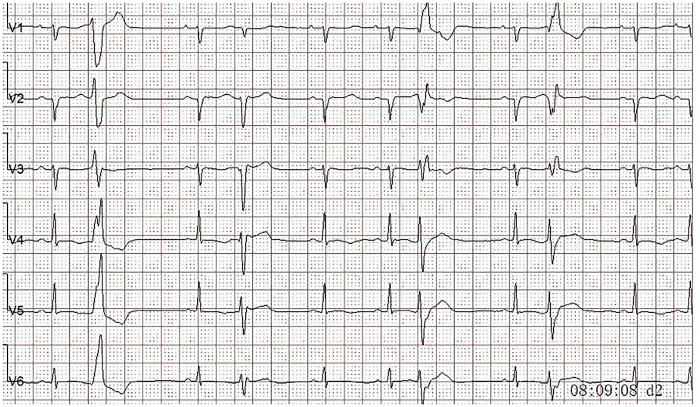
2024.02 Holter monitoring indicates R on T.

**Figure 7:**
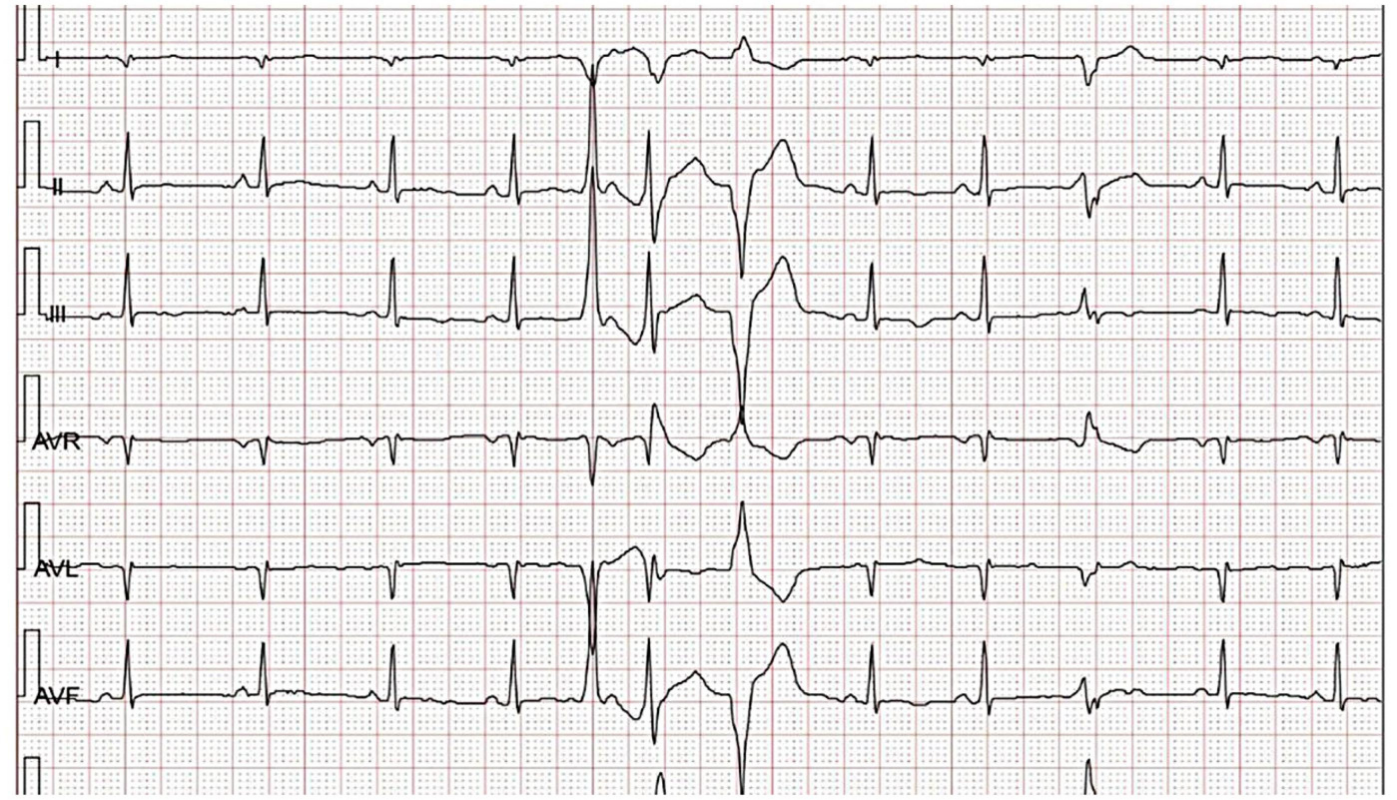
2024.02 Holter monitoring indicates ventricular tachycardia.

A 9-year-old girl was admitted to the hospital in February 2020 due to repeated dizziness for half a year. Cardiac auscultation showed premature beats, and her heart sound was slightly low and dull. Anticardiolipin antibodies and antimyocardial antibodies were negative. High-sensitivity troponin I was measured at 0.35 µg/L (0–0.045 µg/L). Holter monitoring identified premature ventricular beats (Figures 6, 7) (less than 1% of total beats, multi-source, and polymorphic in nature), along with non-sustained ventricular tachycardia (NSVT), abnormal Q waves in leads I and aVL, and low, bidirectional, and inverted T waves in leads V3–V6. Echocardiography (ECHO) revealed a slightly enlarged left ventricle. The left ventricular wall motion was uncoordinated, and the left ventricular ejection fraction (LVEF) was 54%. Cardiac magnetic resonance (CMR) imaging with gadolinium contrast suggested a high likelihood of acute myocarditis, leading to a diagnosis of myocarditis. The patient was treated with intravenous immunoglobulin, glucocorticoids, metoprolol succinate, trimetazidine, mycophenolate mofetil, and perindopril, along with multiple medications for myocardial nutritional support. During follow-up, troponin I levels were still elevated several times.

**Figure 8:**
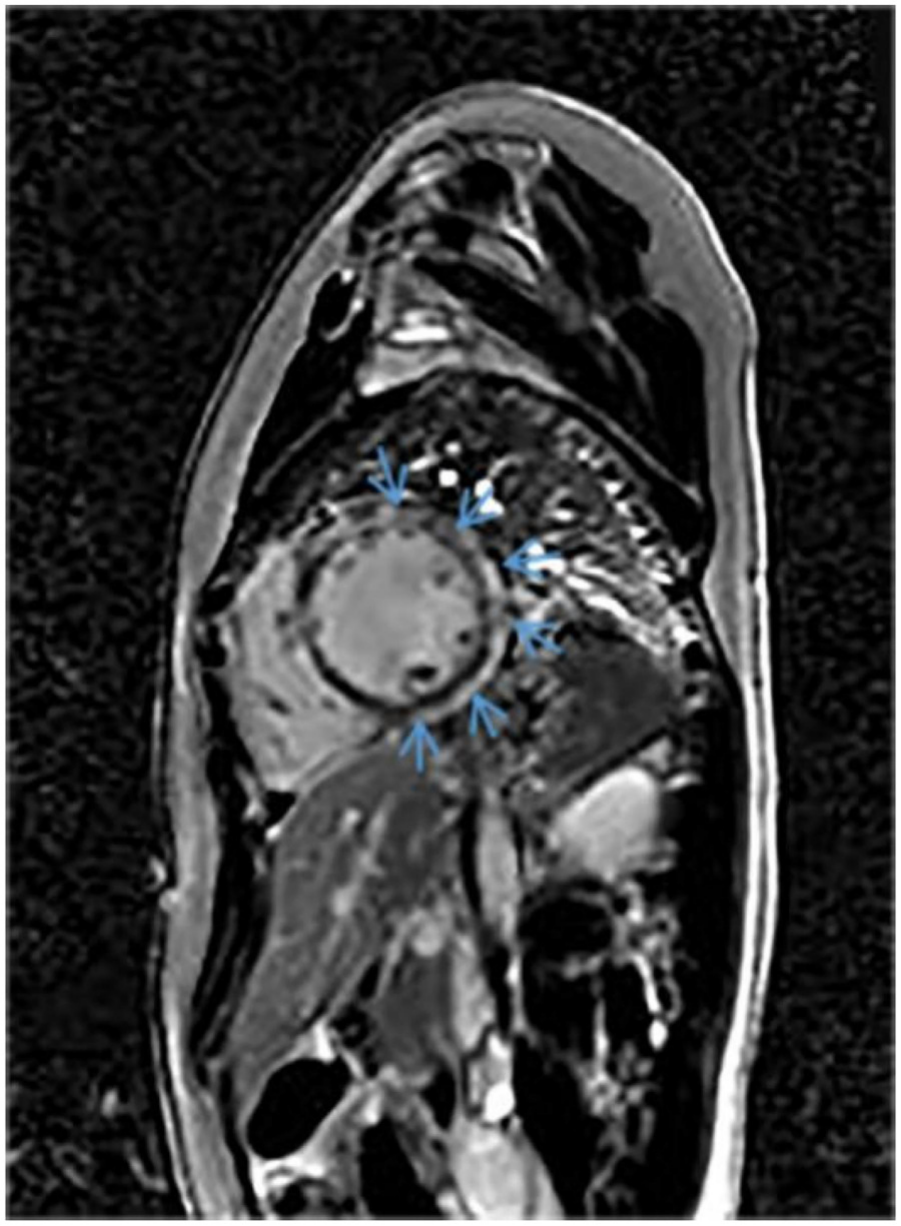
CMR imaging shows changes in the interventricular septum and subepicardial of the LV that form a non-ischemic ring-like pattern on LGE images.

Nearly 1 year after the first admission, CMR imaging showed changes of the interventricular septum and subepicardium of the LV that formed a non-ischemic ring-like pattern in late gadolinium enhancement (LGE) images (Figure 8), and the patient’s LVEF dropped to 46%. Based on a molecular genetic analysis of compound heterozygous variants in *DSP*, the diagnosis was revised to ACM. During the follow-up, we found that the child still had elevated troponin levels on several occasions, decreased LVEF, and increased ventricular premature beats; thus, the treatment plan was adjusted appropriately. Recent re-examination showed that Nt-ProBNP and troponin I levels were normal, and ECHO showed an LVEF of 44%, left ventricular enlargement, and uncoordinated left ventricular wall motion. Holter monitoring showed that ventricular premature beats accounted for 8.9%. The patient’s parents are non-consanguineous and the proband's sister had no discomfort such as dizziness, chest tightness, chest pain, palpitations, syncope, dyspnea, or cardiac arrest and no abnormalities on Holter monitoring or ECHO.

### 3.2 Gene mutation analysis of the proband and family members

**Figure 9:**
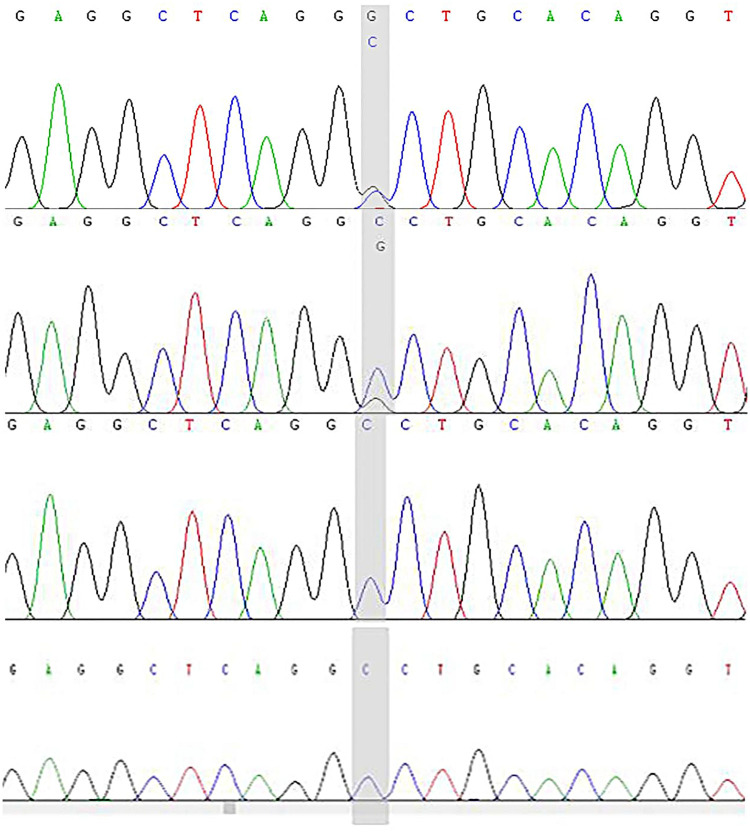
*DSP* gene (c.7964C > G) sequencing map of the proband and her parents. The child has the c.7964C > G (p.Ala2655Gly) heterozygous mutation and the shadow shows the variation points. **(A)** Sanger sequencing map of the proband. **(B)** Sanger sequencing of the proband's father. **(C)** Sanger sequencing of the proband's mother. **(D)** Sanger sequencing of the proband's sister.

**Figure 10:**
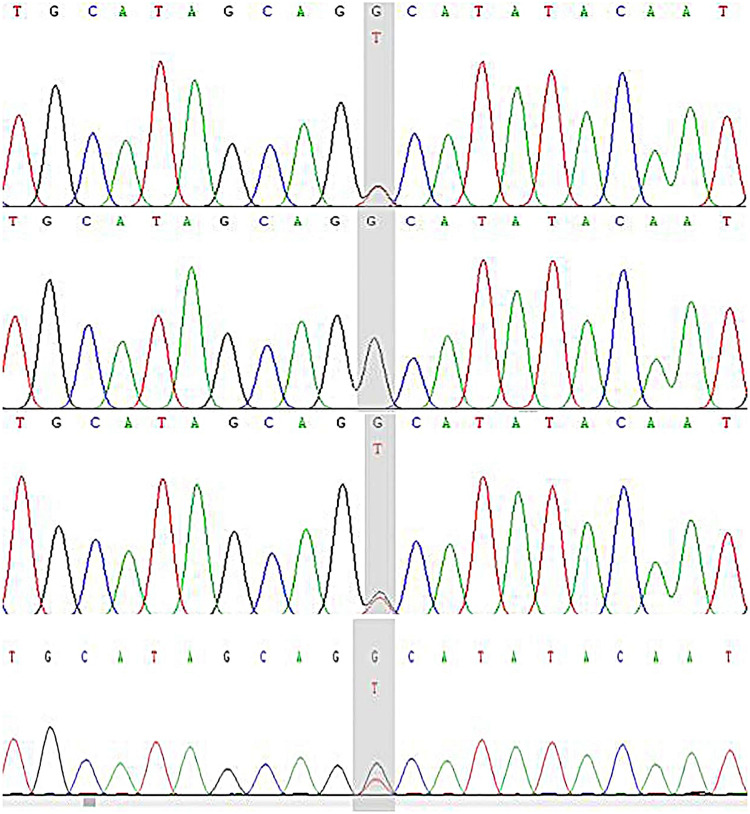
*DSP* gene (c.6785 G > T) sequencing map of the proband and her parents. The child has the c.6785 G > T (p.Gly2262Val) heterozygous mutation and the shadow shows the variation points. **(A)** Sanger sequencing map of the proband. **(B)** Sanger sequencing of the proband's father. **(C)** Sanger sequencing of the proband's mother. **(D)** Sanger sequencing of the proband's sister.

The patient had compound heterozygous variants in the *DSP* gene [NM_004415.4:c.6785G > T(p.Gly2262Val), c.7964C > G(p.Ala2655Gly)], which were inherited from her mother and father, respectively. The patient’s sister also inherited the c.6785G > T (p.Gly2262Val) mutation from their mother (Figures 9, 10). Both variants are located in exon 24 of the *DSP* gene.

**Figure:**
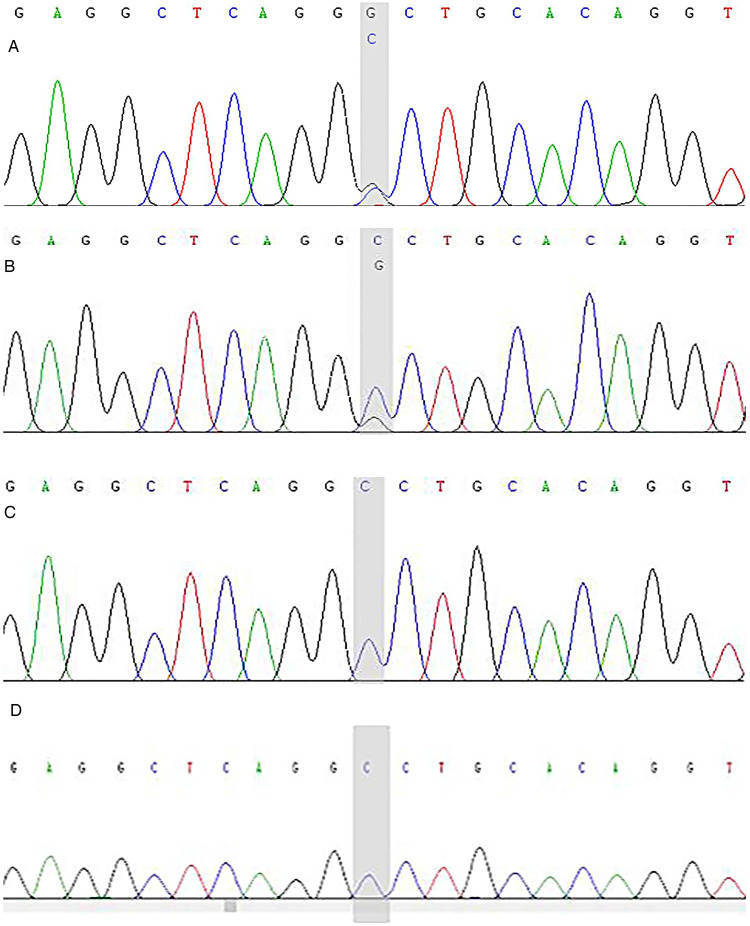


**Figure:**
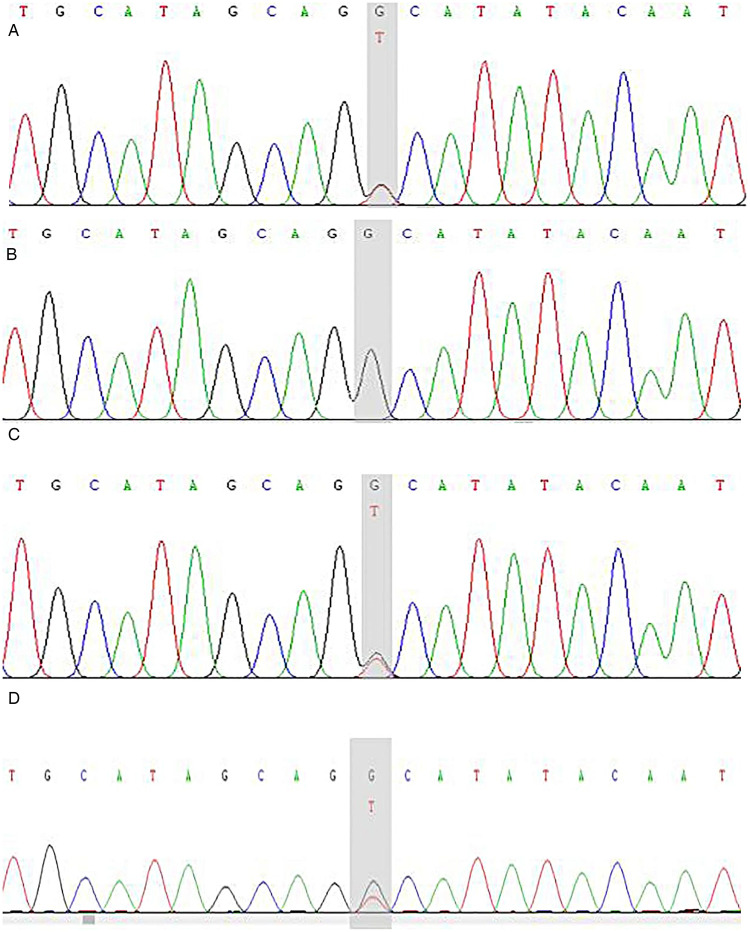


By searching the ClinVar database and gnomAD, we identified the following DSP variants:

1. DSP: NP_001008844.1:c.6785G > A/T (p.Gly2262Val), rs1422731756—Conflicting classifications of pathogenicity: Uncertain significance (1); Likely benign (1); allele frequency in gnomAD: 0.000002.
2. DSP: c.7964C > G (p.Ala2655Gly), rs193922671—Conflicting classifications of pathogenicity: Likely pathogenic (1); Uncertain significance (4); Likely benign (3); allele frequency in gnomAD: 0.00001115.

Based on the analysis of the two mutations using the SIFT, PolyPhen2_HDIV, PolyPhen2_HVAR, LRT, MutationTaster, and MutationAssessor prediction tools, these variants are deleterious. According to the American College of Medical Genetics and Genomics (ACMG) guidelines, both mutations met the criterion of two pieces of supporting evidence (PM2_Supporting + PP3) and can be classified as variants of uncertain significance (VUS).

## 4 Literature review

**Figure 11:**
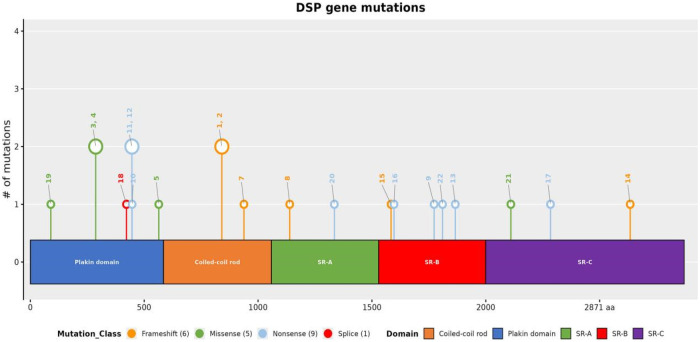
Functional diagram of *DSP* gene variants we collected. The serial numbers correspond to the patient numbers in Supplementary Data Sheet S1.

The literature was systematically searched in the China National Knowledge Infrastructure, Wanfang Database, PubMed, MEDLINE, and Web of Science using the keywords “Myocarditis,” “Arrhythmogenic Ventricular Cardiomyopathy,” “Desmoplakins,” and “Arrhythmogenic Cardiomyopathy” from inception to the present. Among the studies that met the search criteria, there were no Chinese articles and 17 English articles (7–23). The clinical manifestations, auxiliary examinations, and gene mutation sites of the cases in these studies were analyzed. A total of 22 patients with *DSP*-associated ACM that initially presented as myocarditis or had recurrent episodes of myocarditis were collected (Table 1) and summarized (Supplementary Data Sheet S1). We also provide a functional diagram of the *DSP* gene variants we collected (Figure 11).

**Table 1:** Comparison of the clinical characteristics of patients with *DSP* gene mutations.

Clinical feature	Clinical feature details	Patients with mutations (*n*=22)
Gender (%)	Male	11（50.00%）
	Female	11（50.00%）
Mean age (years)		22.55
Clinical manifestations (%)	Exercise triggers	2（9.09%）
	Chest pain	19（86.36%）
	Syncope	2（9.09%）
	Sudden cardiac arrest	1（4.54%）
	Curly hair or palmoplantar keratosis	4（18.18%）
	Dyspnea	2（9.09%）
Electrocardiographic findings (%)	Ventricular tachycardia	7（31.82%）
	Premature ventricular contractions	2（9.09%）
	Ventricular fibrillation	1（4.54%）
	Supraventricular tachycardia	1（4.54%）
	Right bundle branch block	1（4.54%）
	Left bundle branch block	0（0%）
	Low voltage	2（9.09%）
	ST-T changes	9（40.91%）
	Atrial fibrillation	1（4.54%）
	V1–V3 epsilon wave	1（4.54%）
Echocardiography (%)	Structural abnormalities or functional decline of the left ventricle	13（59.09%）
	Structural abnormalities or functional decline of the right ventricle	1（4.54%）
	Structural abnormalities or functional decline of both ventricles	1（4.54%）
CMR (%)	Changes in the LV on LGE images	17（77.27%）
	Changes in the RV on LGE images	1（4.54%）
	Changes in both ventricles on LGE images	2（9.09%）
	Fatty infiltration in both ventricles	1（4.54%）
Troponin (%)	Elevated	22（100%）
Family history (%)	Cardiomyopathy/SCD	11（50.00%）

The sample of 22 patients had a male-to-female ratio of 1:1 and an average age of onset of 22.55 years. The main clinical symptoms were as follows: 19 patients presented with chest pain, two with syncope, two with shortness of breath, one with cardiac arrest, and four with curly hair or palmoplantar keratoderma. The genotypes identified were as follows: 13 patients had heterozygous mutations, nine had nonsense mutations, five had missense mutations, six had frameshift mutations, one had compound heterozygous variants, one had a homozygous mutation, and one had a splice mutation. All the patients showed ECG abnormalities, including seven with ventricular tachycardia, two with premature ventricular contractions, one with ventricular fibrillation (VF), one with supraventricular tachycardia, one with right bundle branch block, two with low voltage, nine with ST-T changes, one with atrial fibrillation, and one with V1–V3 epsilon waves. Echocardiography revealed left ventricular structural abnormalities or reduced function in 13 cases, right ventricular structural abnormalities or reduced function in one case, and biventricular structural abnormalities or reduced function in one case. CMR imaging with both plain scans and contrast enhancement showed left ventricular LGE in 17 patients, right ventricular LGE in one patient, biventricular LGE in two patients, and biventricular fat infiltration in one patient. All the patients had elevated troponin levels. Additionally, 11 patients had a family history of cardiomyopathy and SCD.

## 5 Discussion

In this case study, we report a child with *DSP*-associated ACM who presented with myocarditis as the initial manifestation, with ring-like enhancement on magnetic resonance imaging, frequent ventricular premature contractions and T wave inversion on Holter monitoring, and left ventricular enlargement on ECHO. Genetic testing revealed compound heterozygous variants in the *DSP* gene. However, due to the lack of functional verification assays, these variants were only classified as variants of uncertain significance. The typical clinical manifestations of ACM usually present in adolescence or later. This proband represents the youngest reported patient with *DSP*-associated ACM.

DSP plays an important role in cell adhesion, cytoskeletal dynamics, electrical conduction-repolarization dynamics, and embryonic development (24). The results of a 2022 retrospective multicenter study showed that 91.7% of patients with *DSP* gene variants and left ventricular involvement had “hot phase” symptomatology, and their risk of adverse cardiac events and heart failure was much higher than that of patients who presented with acute myocarditis-like symptoms without *DSP* gene mutations (25). The “hot phase” of *DSP*-associated ACM is defined as the onset of myocarditis-like symptoms, which is characterized by chest pain, elevated troponin levels, and abnormal electrocardiogram findings in patients with normal coronary arteries. Affected individuals or their relatives often have a history of recurrent acute myocarditis, cardiomyopathy, or sudden cardiac death. Scheel et al. identified a group of patients with ACM who presented with myocarditis as an initial presentation to have unique features, including younger age at onset, female sex, left ventricular involvement, and *DSP* genetic variants (26). However, there was no difference in the gender distribution of the patients we collected.

After treatment, the patient initially showed improvement in premature ventricular contractions (PVCs), with the frequency controlled at 1%–3%. However, the 3-year follow-up revealed an increase in PVCs (11.3%) even though troponin levels returned to normal, along with a decrease in LVEF to 44%. This indicates that even after the hot phase, arrhythmias worsened with pharmacological control and were accompanied by heart failure. This patient's 5-year risk for sustained ventricular tachycardia (VT), VF/atrial flutter, cardiac arrest, or SCD was approximately 47%, as assessed by the DSP risk-scoring tool (http://www.dsp-risk.com) developed in 2019. Due to the patient's young age and suboptimal pharmacological control, implanting an implantable cardioverter defibrillator should be considered if episodes of syncope, polymorphic ventricular tachycardia, ventricular aneurysm, or recurrent non-sustained ventricular tachycardia occur in the future. There was no family history of heart disease previously. The patient had no identifiable triggers such as exercise or infection. The difference between the patient and her family members lies in the presence of compound heterozygous variants in the *DSP* gene. It is speculated that the onset probability, age of onset, occurrence of the “hot phase,” and disease prognosis are related to the dose accumulation effect and the degree of damage to the functional domain of the mutated *DSP* gene.

Through a literature review, we found that DSP-associated ACM often presents with chest pain, ventricular tachycardia, left ventricular structural abnormalities or dysfunction, and subepicardial changes in the LV on LGE images, forming a non-ischemic ring-like pattern. The primary structure of desmoplakin consists of three functional domains, i.e., the N-terminal domain (residues 1–1,056), the rod segment (residues 1,057–1,945), and the C-terminal domain (residues 1,946–2,871). In children and adolescents, five mutations have been identified in the N-terminus of DSP, one in the rod segment, and one in the C-terminus. Four mutations associated with malignant arrhythmias (VT/VF) have been located in the N-terminus, while three have been found in the rod segment. The sites associated with ST-T changes were located in the N-terminus in three cases and in the rod segment in three cases. One C-terminal mutation has been associated with atrial fibrillation. No significant difference was observed in the types of mutations identified. Notably, the patients with *DSP* gene mutations located in the N-terminus and rod segments were younger and more prone to malignant arrhythmic events.

Both of the mutations in the proband are located in the C-terminus. The Ala at position 2655 resides in PRD2 of the C-terminal segment. The Gly at position 2262 resides at the end of PRD1 of the B-terminal segment and is extremely highly conserved. The crystal structure of segment B revealed that the glycine residues at the end of PRD1 and PRD3 adopt a backbone conformation available only to glycine, which produces a sharp turn. Thus, the mutation described here, i.e., the substitution of glycine in position 2262 with valine, is likely to alter the B segment and disrupt the desmoplakin–IF interaction. Compound heterozygous variants that both affect C-terminal protein reference datasets (even in different PRDs) can produce additive or synergistic loss of IF anchoring; one allele may destabilize PRD folding, while the other may reduce the binding affinity. Together, these effects produce loss of function and a severe phenotype.

In summary, we have identified two *DSP* variants in ACM. This case underscores the importance of considering ACM in patients with recurrent myocarditis and highlights the role of genetic testing in revealing the underlying cause. Patients with persistently elevated troponin levels, cardiac magnetic resonance imaging showing myocardial inflammation, subepicardial and myocardial LGE, or a family history of myocarditis/cardiomyopathy should be evaluated for ACM, and genetic testing should be performed for diagnostic confirmation and risk stratification. A cautious interpretation may be warranted, potentially classifying these variants as VUS, depending on additional evidence. Early diagnosis of ACM and recognition of the inflammatory phase are critical for patient management, including exercise restriction, risk stratification, disease prevention, close monitoring, and cascade screening of family members.

## Data Availability

The datasets presented in this study can be found in online repositories. The names of the repository/repositories and accession number(s) can be found in the article/Supplementary Material.
