# Supplementary material for: Genotype–phenotype correlations in DSP-associated arrhythmogenic cardiomyopathy that initially presents as myocarditis: a case report and literature review
Source: Front Cardiovasc Med. 2026 May 19;13:1789765. doi: 10.3389/fcvm.2026.1789765 (PMC13226180; doi:10.3389/fcvm.2026.1789765)
Supplement: Supplementary file 1 [file Datasheet1.pdf]

12	13	14	15	16	17
17	18	19	20	21	22
Exon24	Intron	Exon2/Exon3	Exon23	Exon24	Exon24
c.6850C>T	c.1267-2A > G	c.268C>T/c.304C>T	c.4003C>T	c.6328G>C	c.5428C>T
p.Arg2284X		p.Gln90/p.Arg102Cys	p.Gln1335*	p.Ala2110Pro	p.Gln1810Ter
Missense mutation	Variation in splicing	Compound heterozygous missense mutation	Heterozygous nonsense mutation	homozygous missense mutation	Heterozygous nonsense mutation
male	female	female	female	male	male
13 years old	21 years old	18 years old	43 years old	23 years old	23 years old
Chest pain, palpitations, curly hair, mother's curly hair, ACM with SCD as the first appearance	Chest pain was accompanied by radiating pain in both upper limbs for 2 days, and the younger brother had ACM and myocarditis	SCD	There were repeated chest pains and sudden death of the father	Syncope, palpitations, palmoplantar keratosis	Chest pain, acute myocarditis
elevated	elevated	elevated	elevated	elevated	elevated
Not obvious	The ST-T is changed and the T wave is inverted	Ventricular tachycardia, sinus bradycardia, one degree atrioventricular block, prolonged QTc interval	ST segment depression in inferior leads	Atrial fibrillation, epsilon wave, V2-V5T wave inversion, and ectopic right bundle branch block morphology	The T wave was inverted in the lateral leads
Biventricular function was preserved	LVEF 51% and mild hypokinesis of the mid-inferolateral wall	LVEF was 53% and biventricular size was normal	LVEF was normal	LVEF 48%	
impressive amount of subepicardial, circumferential hyperenhancement with a "ring-like" appearance extending from the LV base to apex, Oedema imaging showed areas of high T2 signal intensity overlapping the LV LGE hyperenhanced areas, The 5-month follow-up CMR showed normal biventricular size and function, with the same pattern and extent of myocardial hyperenhancement on	There was subepicardial LGE of the inferior, inferolateral, anterolateral, and anterior LV walls with mild overlying pericardial LGE suggestive of myopericarditis,Right ventricle (RV) was normal in size and systolic function,mildly dilated LV	contiguous mesocardial and epicardial late gadolinium enhancement in a ring-like distribution involving the mesocardium and epicardium.	ring-like pattern of late gadolinium enhancement showing near circumferential involvement of the mesocardium and epicardium	LVEF31% ,RVEF40%,biventricular regional wall motion abnormalities, nonischemic LGE with a ringlike pattern and subepicardial/intramyocardial extension involving all LV segments and part of the RV free wall	a non-dilated LV with low-normal EF, as well as normal RV dimensions and function. T2-weighted images highlighted the presence of mid-wall myocardial edema involving the interventricular septum, where mid-wall LGE,a diffuse circumferential subepicardial LGE involvement of the LV myocardium
			Right ventricular septal endomyocardial biopsy was notable for pathologic adipocyte infiltrate and interstitial fibrosis without evidence of acute myocardial injury, vasculitis, or amyloidosis		
	COVID - 19 Ag Positive			SARS-CoV-2(+)	
elevated				elevated	

7	7	8	9	10	11
11	12	13	14	15	16
Exon11	Exon11	Exon24	Exon24	Exon23	Exon23
c.1339C>T	c.1339C>T	c. 5596C > T	c.7899 dup	c.4751_4752del	c.4789 G > T
		Q1866X	p.Thr2634Tyrfs * 10	p.Ala1584Valfs * 42	p.Glu1597 *
Dominant nonsense mutation	Nonsense mutation	Nonsense heterozygous mutation	Heterozygous frameshift mutation	Heterozygous frameshift mutation	Heterozygous nonsense variants
elder sister og 9	female	male	male	female	female
19 years old	38 years old	36 years old	20 years old	16 years old	24 years old
Chest pain recurred after the diagnosis of ALVD	Acute chest pain developed two weeks after delivery and her mother had ALVD	Chest pain, cold, dyspnea, myocarditis, sister had ACM	Chest pain, recurrent episodes of myocarditis	left dominant arrhythmogenic cardiomyopathy and pain in the right chest, shoulder and back for 5 days	Chest pain
elevated	elevated	elevated	elevated	elevated	elevated
flat T waves		Sustained ventricular tachycardia、atrioventricular conduction disorders, right bundle branch block and superior axis block, low voltages, a delay in the activation of the QRS terminal of the inferior face and V4-V6 and transtornodelaconducci on the atrioventricula	normal	polymorphic ventricular ectopy and nonsustained runs of ventricular tachycardia.,minimal ST-segment elevation in the inferior leads	VF and VT
progressive LV impairment documented during the follow-up			normal	normal	
widespread LGE pattern		Mild LV systolic dysfunction	Myocardial inflammation and LV changes in LGE	significant subepicardial LGE in the left ventricle basal, mid and apical anterior, septal, inferior, and inferolateral wall regions, and myocardial edema involving the interventricular septum	LVEF: 43%, diastolic slightly dilated left ventricle, subepicardial late gadolinium enhancement (LGE) and pericardial effusion
		Steatosis/fibrosis			interstitial edema , There was myocardial hypertrophy in the right ventricle with increased adipose tissue and myocardial fibrosis in the LV

3	4	5	6	7	7
5	6	7	8	9	10
Exon13		Exon20	Exon23	Exon23	Exon11
c.1691C>T	DSP	c.2811_2812dupAT	c.3415_3417delTATinsG	c.5318delT	c.1339C>T
p.Thr564Lle		p.Ser938Tyrfs*	p.Tyr1139GlyfsX10		p.Gln447Ter
Heterozygous missense		Heterozygous frameshift mutation	Heterozygous frameshift mutation	Nonsense mutation	Dominant nonsense mutation
male	female	female	male	male	younger brother of 9
10 years old	17 years old	47 years old	24 years old	20 years old	14 years old
Chest pain, palmoplantar keratosis, sparse hair, hypodontia, brittle nails	Chest pain, myocarditis	Acute myocarditis, syncope, curly hair, palmoplantar keratosis	Mountain bikers, colds, chest pain, sweating, dizziness, difficulty breathing	Acute myocarditis was suspected with recurrent chest pain and biventricular dysfunction	Chest pain recurred after the diagnosis of ALVD
elevated	elevated	elevated	elevated	elevated	elevated
Multiple isolated ventricular premature beats, V1 epsilon wave	The T wave is inverted	low voltages in inferior and lateral leads with depolarization abnormalities.She had sustained ventricular tachycardia	rare ventricular ectopic beats (82 in 24 hours) but no ventricular tachycardia		dynamic T wave inversion in inferolateral leads
The infundibulum of RV was dilated and LVEF was normal		LVEF43%、a moderately depressed left ventricular ejection fraction (43%) with diffuse hypokinesia. The right ventricle was normal	LVEF 65% with hypokinesis of the mid and apical lateral LV segments	Mild LV dilatation, LVEF 45%	The left ventricle was progressively damaged and LVEF decreased
Acute left ventricular myocarditis with late gadolinium enhancement in RV	abnormal T1 and T2 signal in the basal left ventricle on late gadolinium enhancement images and T2 maps respectively	extensive sub-epicardial fibrosis primarily in the inferior, posterior, lateral, and anterior left ventricular walls	normal LV and RV sizesand function, multiple areas of fibrofatty replacement involving the anterior, lateral and inferior LV segments, and the basal inferior RV free wall.areas of fatty replacement within the LV myocardium		a widespread LGE pattern in LV

The literature	1	1	2	2
Patient Number	1	2	3	4
Exons/introns	Exon18	Exon18	Exon7	Exon7
Nucleic acid changes	c.2521_2522del	c.2521_2522del	c.860A>G	c.860A>G
Changes in protein	p.Gln841Aspfs*9	p.Gln841Aspfs*9	p.Asn287Ser	p.Asn287Ser
Type of mutation	Heterozygous frameshift mutation	Heterozygous frameshift mutation	missense heterozygous mutation	missense heterozygous mutation
Gender	male	younger brother of 1	female	younger sister of 3
Age of onset	18 years old	17 years old	20 years old	15 years old
Clinical manifestations and family history	Chest pain after the flu, myocarditis recurred one year later	Tonsillitis with chest pain while playing soccer	Repeated chest pain	There were two episodes of myocarditis with chest pain
cTn	elevated	elevated	elevated	elevated
Electrocardiogram	ST-T was slightly elevated ， NSVT after 1 year	ST-elevation in inferior leads	Sinus tachycardia, PVC, NSVT,no ischemic changes	
echocardiography	LV with reduced inferolateral wall motion.，had normal dimensions with LVEF of 46%,and reduced to 53% after 1 year		LV was normal	The LVEF was normal
CMR	comprehensive inflammatory, subepicardial changes of the LV in,CMR during 1 year follow-up still depicts extensive subepicardial scars of the LV but no myocardial oedema	extensive subepicardial non-ischaemic scars of the LV withinflammation and an LVEF of 52%.	edema and/or increased interstitial space (previous fibrosis), while the elevation of myocardial T2 was consistent with edema predominantly involving the middle septum. The LGE image showed a non-ischemic ring-shaped pattern involving the subepicardium and midmyocardium in the anterior middle and apical, septum, and inferior segments	LGE was identified in the mid-inferior septum and inferior wall.
Histology	No interstitial fibrosis was detected, and only subendocardial fibrosis was seen			
Etiology				
BNP				

（1）Kissopoulou A, Fernlund E, Holmgren C, Isaksson E, Karlsson JE, Green H, et al. Monozygotic twins with myocarditis and a novel likely pathogenic desmoplakin gene variant. ESC Heart Fail. (2020) 7:1210 1216. doi: 10.1002/ehf2.12658.

（2）Koike H, Idris A, Berger J, Cheng VY, Sengupta J, Cavalcante JL. Acute Myocarditis as the Initial Presentation of Desmoplakin Mutation - Broadening the Differential Diagnosis. Arq Bras Cardiol. (2024) 121:e20230541. Portuguese, English. doi: 10.36660/abc.20230541.

（3）Keller DI, Stepowski D, Balmer C, Simon F, Guenthard J, Bauer F, et al. De novo heterozygous desmoplakin mutations leading to Naxos-Carvajal disease. Swiss Med Wkly. (2012) 142:w13670. doi: 10.4414/smw.2012.13670.

（4）Gowda PC, Gasperetti A, Zimmerman SL. NASCI case of the month: desmoplakin cardiomyopathy masquerading as acute myocarditis. Int J Cardiovasc Imaging. (2024) 40:1397 1398. doi: 10.1007/s10554-024-03112-z.

（5）Efthimiadis G, Zegkos T, Meditskou S, Karamitsos T, Manolakos E, Papoulidis I, et al. A novel desmoplakin mutation associated with left dominant arrhythmogenic cardiomyopathy and cutaneous phenotype. Hellenic J Cardiol.(2021) 62:95 98. doi: 10.1016/j.hjc.2020.04.014.

（6）Reichl K, Kreykes SE, Martin CM, Shenoy C. Desmoplakin Variant-Associated Arrhythmogenic Cardiomyopathy Presenting as Acute Myocarditis. Circ Genom Precis Med. (2018)11:e002373. doi: 10.1161/CIRCGEN.118.002373.

（7）Lopez-Ayala JM, Pastor-Quirante F, Gonzalez-Carrillo J, Lopez-Cuenca D, Sanchez-Munoz JJ, Oliva-Sandoval MJ, et al. Genetics of myocarditis in arrhythmogenic right ventricular dysplasia. Heart Rhythm.(2015) 12:766 73. doi: 10.1016/j.hrthm.2015.01.001.

（8）Navarro-Manchón J, Fernández E, Igual B, Asimaki A, Syrris P, Osca J, et al. Left dominant arrhythmogenic cardiomyopathy caused by a novel nonsense mutation in desmoplakin. Rev Esp Cardiol. (2011) 64:530 4. doi: 10.1016/j.recesp.2010.10.020.

（9）Morales JP, Spaccavento A, Guerchicoff M, Burgos L, Costabel JP, Cingolani E, et al. Diagnosis of Arrhythmogenic Cardiomyopathy in a Young Patient With Recurrent Myocarditis: The Importance of Genetic Testing. Am J Cardiol. (2025) 242:37 40. doi: 10.1016/j.amjcard.2025.01.034.

（10）Fanucci V, Chauhan D, Chaudhuri NR, Nayak HM, Kohli U. A novel familial pathogenic desmoplakin (*DSP*) gene mutation (c.4751_4752del) presenting as "hot-phase" left-dominant arrhythmogenic cardiomyopathy. Future Cardiol. (2025) 21:473 478. doi: 10.1080/14796678.2025.2500835.

（11）Westphal DS, Krafft H, Biller R, Klingel K, Gaa J, Mueller CS, et al. Myocarditis or inherited disease? - The multifaceted presentation of arrhythmogenic cardiomyopathy. Gene. (2022) 827:146470. doi: 10.1016/j.gene.2022.146470.

（12）Pătru AE, Onciul S, Sturzu A, et al. Acute Myocarditis-Like Episode in a Curly-Haired Young Boy-Red Flags for Familial Arrhythmogenic Cardiomyopathy. Diagnostics (Basel). (2020) 10:651. doi: 10.3390/diagnostics10090651.

（13）Rezaei Bookani K, Minga I, Wodskow J, et al. A case series of desmoplakin cardiomyopathy: a mimic of viral myocarditis. Eur Heart J Case Rep. (2022) 10:651. doi: 10.3390/diagnostics10090651.
（14）Srinivas RE, Wright LK, Nandi D, Hayes EA. A case for genetic testing: Arrhythmogenic cardiomyopathy presenting as myocarditis. Ann Pediatr Cardiol. (2024) 17:55 58. doi: 10.4103/apc.apc_122_23.
（15）Sanford CB, Fan J, Hua Y, Nikolaidis L, Edmister W, Payne S,et al. Case Illustration of the Natural History of Left Dominant Arrhythmogenic Cardiomyopathy. Ochsner J. (2024) 24:62 66. doi: 10.31486/toj.23.0057.
（16）Giannoni A, Modena M, Montuoro S, Bonanni F, Grigoratos C, Barison A, et al. A Novel Homozygous Mutation of the Desmoplakin Gene With Biventricular Arrhythmogenic Cardiomyopathy. JACC Case Rep. (2025) 30:103688. doi: 10.1016/j.jaccas.2025.103688.
（17）RUBINO M, SCATTEIA A, FRISSO G, et al. Imaging the “Hot Phase” of a Familiar Left-Dominant Arrhythmogenic Cardiomyopathy[J]. Genes, 2021, 12(12): 1933.
